# Predicting psychiatric risk: IgG N-glycosylation traits as biomarkers for mental health

**DOI:** 10.3389/fpsyt.2024.1431942

**Published:** 2024-11-22

**Authors:** Yinchun Lv, Yulin Chen, Xue Li, Qiaorong Huang, Ran Lu, Junman Ye, Wentong Meng, Chuanwen Fan, Xianming Mo

**Affiliations:** ^1^ Department of Neurology, Laboratory of Stem Cell Biology, Frontiers Science Center for Disease-related Molecular Network, West China Hospital, Sichuan University, Chengdu, Sichuan, China; ^2^ Department of Occupational and Environmental Health, West China School of Public Health and West China Fourth Hospital, Sichuan University, Chengdu, China; ^3^ West China-PUMC C. C. Chen Institute of Health, West China School of Public Health, and West China Fourth Hospital, Sichuan University, Chengdu, China; ^4^ Department of Gastrointestinal, Bariatric and Metabolic Surgery, Research Center for Nutrition, Metabolism & Food Safety, West China-PUMC C.C. Chen Institute of Health, West China School of Public Health and West China Fourth Hospital, Sichuan University, Chengdu, China; ^5^ Department of Oncology and Department of Biomedical and Clinical Sciences, Linköping University, Linköping, Sweden

**Keywords:** IgG, N-glycosylation, psychiatric disorders, Mendelian randomization, mental health

## Abstract

**Background:**

Growing evidence suggests that chronic inflammation, resulting from intricate immune system interactions, significantly contributes to the onset of psychiatric disorders. Observational studies have identified a link between immunoglobulin G (IgG) N-glycosylation and various psychiatric conditions, but the causality of these associations remains unclear.

**Methods:**

Genetic variants for IgG N-glycosylation traits and psychiatric disorders were obtained from published genome-wide association studies. The inverse-variance-weighted (IVW) method, MR-Egger, and weighted median were used to estimate causal effects. The Cochran’s Q test, MR-Egger intercept test, leave-one-out analyses, and MR-PRESSO global test were used for sensitivity analyses.

**Results:**

In the Psychiatric Genomics Consortium (PGC) database, genetically predicted IGP7 showed a protective role in schizophrenia (SCZ), major depressive disorder (MDD), and bipolar disorder (BIP), while elevated IGP34, and IGP57 increased SCZ risk. High levels of IGP21 were associated with an increased risk of post-traumatic stress disorder (PTSD), while elevated levels of IGP22 exhibited a causal association with a decreased risk of attention-deficit/hyperactivity disorder (ADHD). No causal relationship between IgG N-glycan traits and autism spectrum disorder (ASD) and no evidence of reverse causal associations was found.

**Conclusion:**

Here, we demonstrate that IgG N-glycan traits have a causal relationship with psychiatric disorders, especially IGP7’s protective role, offering new insights into their pathogenesis. Our findings suggest potential strategies for predicting and intervening in psychiatric disorder risk through IgG N-glycan traits.

## 1 Introduction

The widespread occurrence of psychiatric disorders among millions globally profoundly impacts individuals’ well-being and poses significant challenges to effective treatment and management (1–3). The diverse etiologies and complex biological mechanisms underlying psychiatric disorders have hindered our understanding of their pathogenesis, leading to a scarcity of effective and long-lasting treatments (4, 5). Therefore, there is an urgent need to delve deeper into the potential mechanisms of these disorders, in order to enhance our understanding of their pathogenesis and address the current limitations in treatment options for psychiatric disorders.

The crosstalk between the immune and nervous systems is received increasing attention across a diverse range of psychiatric diseases. The central nervous system (CNS) is regulated by immune processes that maintain homeostasis, enhance resilience, and preserve brain reserve (6). Increasing evidences indicated chronic inflammation plays a significant role in the development and progress of psychiatric disorders (7–9). Chronic inflammation can have many adverse effects on brain function, as it can cause neurotoxicity and neurodegeneration. For example, the production of cytokines during inflammation can lead to neurotoxic effects by increasing the production of reactive oxygen species, reducing monoamine transmission, and potentiating glutamatergic transmission (10, 11). Hence, it is imperative to identify the key factors involved in the inflammatory processes contributing to the development of psychiatric disorders and to maintain a suitable balance of inflammation to ensure the optimal function of the CNS.

Many studies have attempted to identify key elements of immune regulation in the pathogenesis of psychiatric disorders. However, suitable key factors have not been found. Immunoglobulin G (IgG) is the most abundant antibody subclass in the human circulatory system. Blood levels of IgG increase in individuals with acute or chronic inflammation, inflammatory disorders, and autoimmune diseases (12, 13). IgG could bind to immune cells which expressed Fc receptors for IgG (FcγR), enabling it to induce an inflammatory response in peripheral or central immune cells. When N-glycans are connected to the conserved asparagine 297 in the IgG Fc region, they function as a regulatory switch, balancing the pro-inflammatory and anti-inflammatory responses of IgG (14). Alterations in N-glycosylation components of IgG, including galactose, fucose, sialic acid, and bisecting GlcNAc, modify the pro- and anti-inflammatory properties of IgG (15–18). Accumulating evidence has indicated that alterations in serum N-glycans are associated with different psychiatric disorders, including schizophrenia (SCZ) (19), major depressive disorder (MDD) (20), bipolar disorder (BIP) (21), post-traumatic stress disorder (PTSD) (22), attention-deficit/hyperactivity disorder (ADHD) (23), and autism spectrum disorder (ASD) (24). These findings suggest the presence of unique IgG N-glycosylation patterns among different types of psychiatric disorders. IgG N-glycosylation may influence the nervous system through the following pathways, thereby leading to psychiatric disorders. Increased fucosylation of IgG reduces its binding to FcγRIIIa on natural killer (NK) cells, diminishing antibody-dependent cellular cytotoxicity (ADCC), while increased sialylation enhances anti-inflammatory activity by promoting the binding to dendritic cell-specific ICAM-3-grabbing non-integrin (DC-SIGN) (25, 26). These changes can result in the increased production of pro-inflammatory cytokines like TNF-α and IL-6, and a decrease in anti-inflammatory cytokines. This can provoke leakage of the blood–brain barrier (BBB) and extravasation of serum proteins, including IgG or IgG N-glycosylation, into the brain (27, 28). IgG N-glycosylation can enter into the brain and bind to Fc receptors which are expressed on microglia cells (29, 30). Activation of microglia through Fc receptors can induce local inflammation within the brain which may lead to synthesis of cytokines, neurological dysfunction, and behavioral effects (29, 31). In the hippocampus, FcγRII is expressed on parvalbumin-GABAergic expressing interneurons localized at the pyramidal cells layer (32). The hippocampus, crucial for emotion, learning, and memory, is implicated in depression and shows IgG deposition alongside activated microglia in the cortex, striatum, hypothalamus, substantia nigra, and cerebellum (33). Additionally, in the brain, inflammatory cytokines can reduce the availability and release of dopamine in the basal ganglia, such as the striatum, while simultaneously increasing the levels of the excitatory amino acid glutamate. This increase in glutamate activity can be further amplified by the activation of the kynurenine pathway. These alterations in neurotransmitter metabolism affect multiple brain regions, leading to disruptions in neurocircuits that regulate motivation, motor activity, and sensitivity to threats and loss. Consequently, these circuit-based changes contribute to symptoms such as anhedonia, psychomotor slowing, anxiety, arousal, and heightened alarm (34). Both underscore the significant role of IgG N-glycosylation in the inflammatory processes associated with the development of psychiatric disorders. Notably, there are no therapeutic targets for IgG N-glycosylation in the treatment of psychiatric disorders. Due to the limited number of studies on IgG N-glycosylation and psychiatric disorders, establishing a causal relationship between these two is essential.

Mendelian randomization (MR) is a groundbreaking tool that utilizes genetic variants as instrumental variables (IVs) to visualize causal relationships between exposures and outcomes of interest (35). MR can avoid reversing exposure-outcome associations by concentrating solely on the genetically regulated component of exposures. Since genotypes remain largely unchanged from conception (36), the evaluations obtained from MR are not influenced by confounders, ensuring more accurate and reliable results. Therefore, we performed a two-sample bidirectional MR analysis using summary data from GWAS to assess the causal association psychiatric disorders including SCZ, MDD, BIP, PTSD, ADHD, and ASD on the risk of IgG N-glycosylation traits, as well as the causal role of IgG N-glycosylation traits in these disorders.

## 2 Methods

### 2.1 Study design

**Figure 1:**
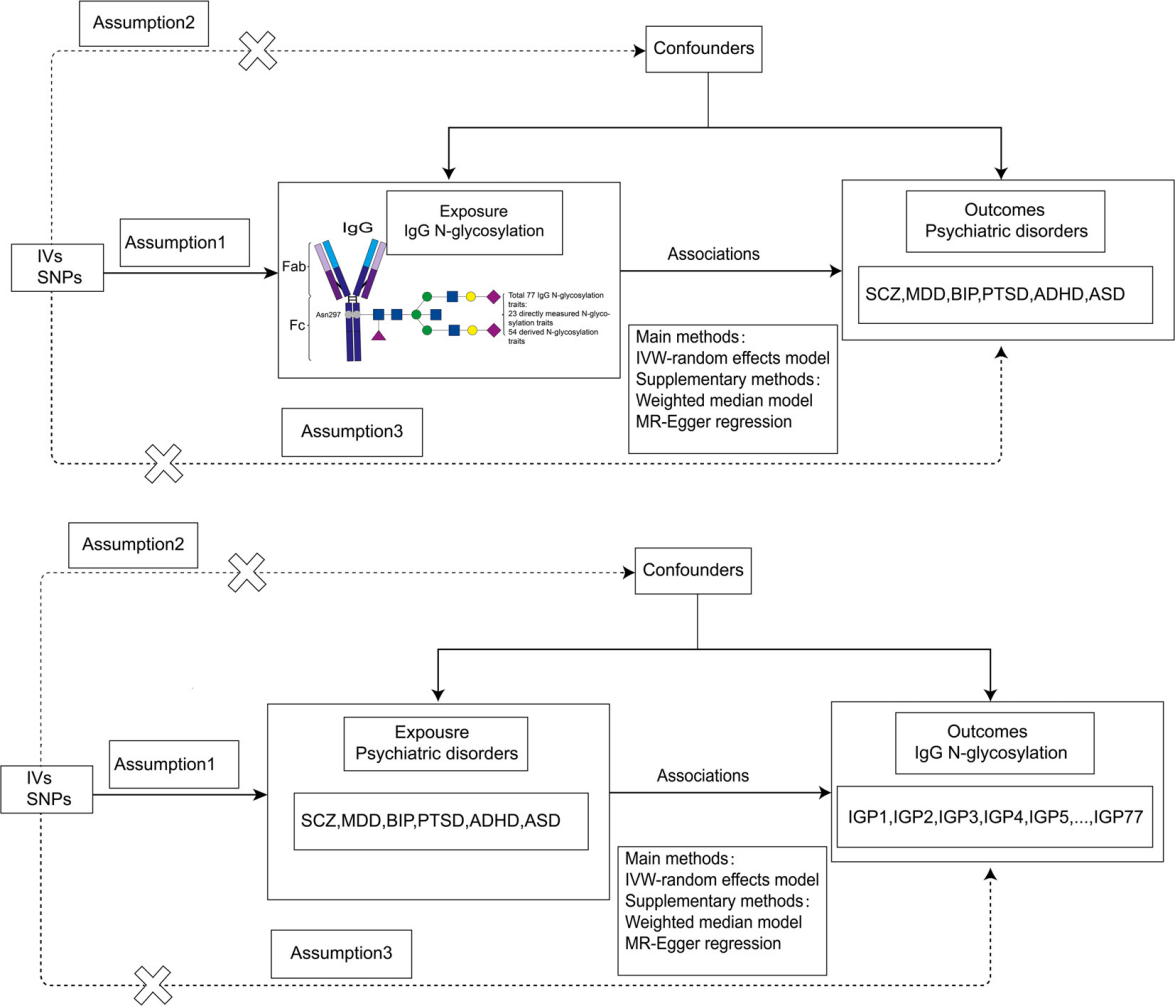
Flowchart of our bidirectional two-sample MR analysis. IVs, instrumental variables; MR, Mendelian Randomization; SNPs, single-nucleotide polymorphisms; IVW, inverse-variance weighted. SCZ, schizophrenia; MDD, major depression; BIP, bipolar disorder; PTSD, post-traumatic stress disorder; ADHD, attention-deficit/hyperactivity disorder; ASD, autism spectrum disorder.

A brief overview of our bidirectional MR analysis is presented in **Figure 1**. In this study, we performed a two-sample bidirectional MR analysis to examine the bidirectional causal effect between IgG N-glycosylation traits and psychiatric disorders (SCZ, MDD, BIP, PTSD, ADHD, ASD). All MR analyses in our study needed to satisfy three basic assumptions:(1) instrumental variables are strongly correlated with exposures, (2) instrumental variables are independent of confounding factors, (3) instrumental variables influence outcomes only through exposure.

### 2.2 Sources of data on the N-glycosylation of IgG

The data used for the summary-level GWAS analysis of IgG N-glycosylation traits was sourced from the largest meta-analysis conducted on 8090 individuals of European descent (37). Summary statistics from GWAS were accessible for a total of 77 IgG N-glycosylation traits, which comprised 23 traits directly measured through ultraperformance liquid chromatography (UPLC) (IGP1-23) and 54 traits derived from the data (IGP24-77). In the directly measured traits using ultraperformance liquid chromatography (UPLC), each peak predominantly corresponds to a significant diantennary complex N-glycan structure. These structures may display distinct features, including core-fucose, bisecting N-acetylglucosamine (GlcNAc), terminal galactose, and terminal sialic acids on the antennae. On the other hand, the derived N-glycosylation traits characterize the quantities or proportions of specific groups of glycans that share particular structural characteristics (Additional File 1; **Supplementary Table S1**).

### 2.3 Sources of data on psychiatric disorders

The GWAS summary statistics for ADHD, ASD, BIP, MDD, PTSD, and SCZ were extracted from the Psychiatric Genomics Consortium (PGC) website (https://www.med.unc.edu/pgc/results-and-downloads/). PGC stands as the most expansive consortium in the annals of psychiatry, spearheading the most impactful meta- and mega-analyses of genome-wide genomic data about psychiatric disorders. The GWAS summary datasets, specific to individuals of European ancestry, including SCZ (38)(33,604 cases and 58,113 controls), MDD (39)(246,363 cases and 561,190 controls), BIP (40)(41,917 cases and 37,1549 controls), PTSD (41)(23,212 cases and 151,447 controls), ADHD (42)(20,183 cases and 35,191 controls), and ASD (43)(18,381 cases and 27,969 controls). These datasets were derived from comprehensive genome-wide meta- or mega-analyses. More details are shown in **Table 1**.

**Table 1:** Characteristics of included genome-wide association studies for MR analysis.

Phenotype	Consortium	Cases	Controls	Total	Population	PubMedIDlink
IgG N-glycans	NA	NA	NA	8090	European	32128391
ADHD	PGC	20,183	35,191	55,374	European	30478444
ASD	PGC	18,381	27,969	46350	European	30804558
MDD	PGC	246,363	561,190	807,553	European	30718901
PTSD	PGC	23,212	151,447	174,659	European	31594949
BIP	PGC	41,917	371,549	413,466	European	34002096
SCZ	PGC	33,604	58,113	91,717	European	35396580

Detailed information of the studies and datasets used for Mendelian randomization analyses. SCZ, schizophrenia; MDD, major depression; BIP, bipolar disorder; PTSD, post-traumatic stress disorder; ADHD, attention-deficit/hyperactivity disorder; ASD, autism spectrum disorder.

### 2.4 Determination of exposure

In this MR study, SNPs associated with IgG N-glycosylation traits at a genome-wide significance level (*P* < 5E-8) from the summary-level GWAS were selected. We excluded SNPs in linkage disequilibrium (LD, r^2^ < 0.001 within a 10 Mb window). The R^2^ and F statistic for each SNP were calculated using the formulas: R^2^ = 2 × EAF × (1−EAF) × β^2^ and F statistic = R^2^ × (N−2)/(1−R^2^). SNPs with F statistics > 10 were recommended for subsequent MR analysis to avoid using weak genetic instruments. We further used online tools PhenoScannerV2 (http://www.phenoscanner.medschl.cam.ac.uk/) to detect potential SNPs associated with the selected ones, which may affect results (i.e., autoimmune, rheumatoid arthritis, smoking, education, drinking, obesity, etc.) (44). Additionally, we excluded the IgG N-glycosylation traits with less than 3 SNPs to meet the minimum requirement of the number of SNP for some MR sensitivity analyses (45). Finally, we used 60 IgG N-glycosylation traits and a total of 325 SNPs for MR analysis (**Supplementary Table S2**).

To investigate the causal effect of psychiatric disorders on IgG N-glycosylation traits, a genome-wide significance level (*P* < 5E-8) was used. Other selection procedures were the same as those for IgG N-glycosylation traits. There were some differences in calculating R^2^ and F values of MDD as it does not sample volume. So we used 2 × ((β)^2) × EAF × (1-EAF) and (β)^2/(se)^2) to calculate respectively. Selected SNPs can be found in **Supplementary Table S3**. Since there were not enough significant SNPs for PTSD, and ASD, we did not include them in the reverse MR analysis. The excluded SNPs as potential confounding factors of IgG N-glycosylation and psychiatric disorders can be found in **Supplementary Table S4**.

### 2.5 Mendelian randomization analysis

To assess the causal effect of IgG N-glycosylation traits on the risk of psychiatric disorders, the random effect inverse variance weighted (IVW) method was employed to estimate. Additionally, MR-Egger and weighted median methods were employed to enhance IVW estimates, as they offer robust estimates in a broader range of scenarios, albeit with less efficiency (i.e., wider confidence intervals [CIs]). All results were presented as odds ratios (ORs) and corresponding 95% CIs for psychiatric disorder outcomes per genetically predicted increase in IgG N-glycosylation levels.

### 2.6 MR sensitivity analyses

Furthermore, comprehensive sensitivity analyses were conducted to estimate potential violations of model assumptions in the MR analysis. We performed Mendelian randomization pleiotropy residual sum and outlier (MR-PRESSO) analysis, along with leave-one-out analysis to detect outlier instrumental variables (46). Instrumental variables identified as outliers by the MR-PRESSO test were systematically removed to mitigate the impact of horizontal pleiotropy. Cochran’s Q test was employed to assess heterogeneity across individual causal effects, and MR-Egger regression was conducted to evaluate the directional pleiotropy of instrumental variables (47).

### 2.7 Data analysis

The Bonferroni correction for multiple testing was conducted to correct *P* values. A *P* value less than 1.39E-4 (0.05/360) was considered as strong evidence of a causal association. All analyses were carried out using the TwoSample MR (version 0.5.6) and MRPRESSO (version 1.0) packages in R software (version 4.2.0).

## 3 Results

### 3.1 The selection of IgG N-glycosylation genetic instruments

To explore the bidirectional causal relationship between IgG N-glycosylation traits and psychiatric disorders, we employed genetic variants strongly associated with these traits, derived from the most extensive GWAS meta-analysis currently available. The selected SNPs, with F-statistics ranging from 30 to 1499 (**Supplementary Table S2 and S3**), demonstrated robust effects, avoiding weak instrument bias. Consequently, a total of 325 SNPs associated with 60 IgG N-glycosylation traits were identified and used as exposures in the forward MR analysis, while 148, 42, 43, and 44 SNPs were selected for SCZ, MDD, BIP, and ADHD, respectively, in the reverse MR analysis. (**Supplementary Table S2, S3**).

### 3.2 The causal effect of IgG N-glycosylation traits on psychiatric disorders

**Figure 2:**
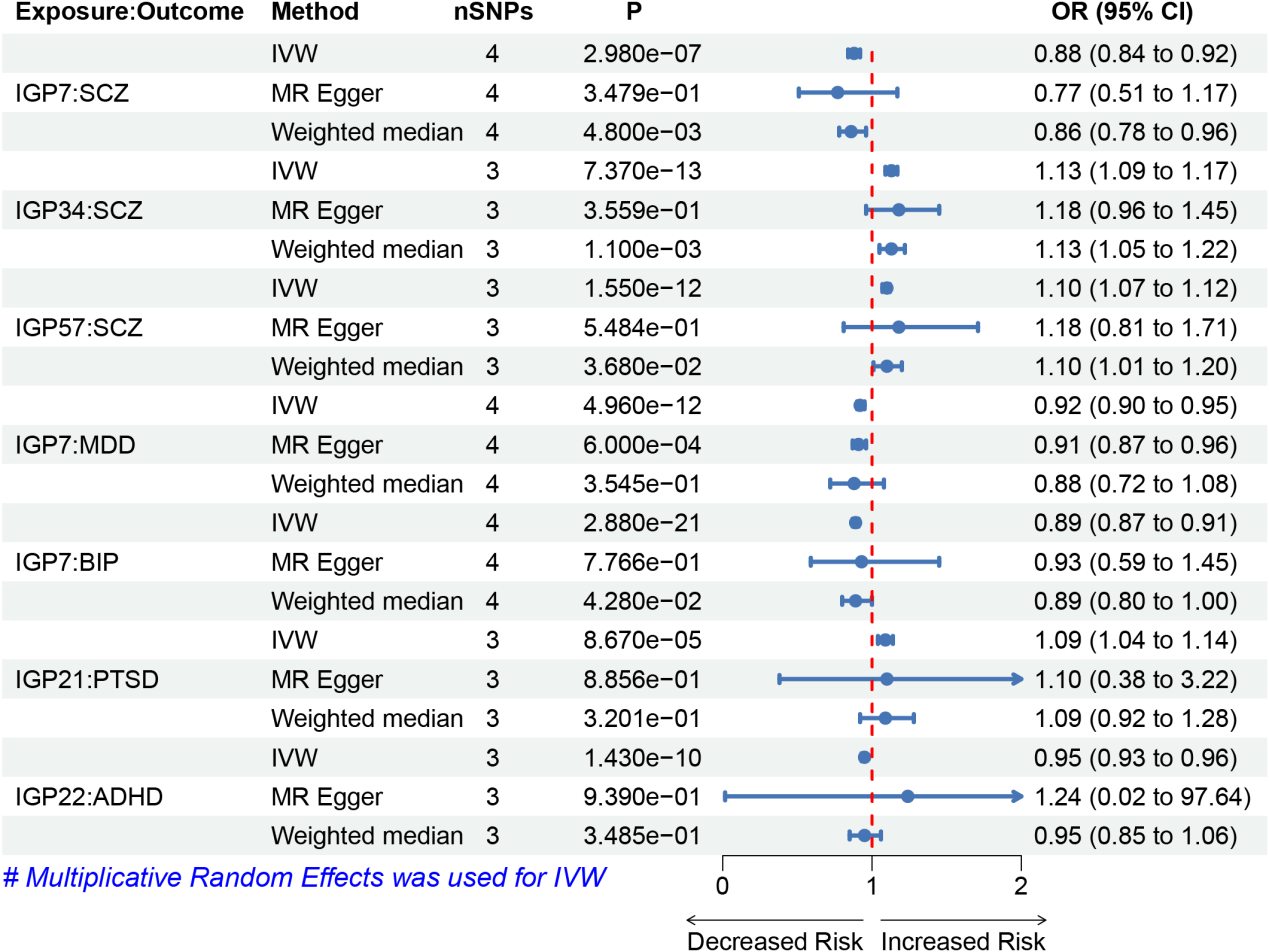
Forest plots of causal effects for IgG N-glycosylation traits on psychiatric disorders. Summary of the MR result derived from the inverse-variance weight, MR-Egger, and weighted median method. The OR was estimated using different MR method. MR, Mendelian Randomization; SNPs, single-nucleotide polymorphisms; OR, odds ratio.

To investigate the causal impact of IgG N-glycosylation traits on psychiatric disorders, we used the IVW method as the primary MR approach. We observed a significant causal association between genetically predicted IgG N-glycosylation traits IGP7 and a reduced risk of SCZ, MDD, and BIP (**Figure 2**). Each standard deviation (SD) increase in genetically determined IGP7 was associated with a 12% decreased risk of SCZ (OR = 0.88, 95% CI = 0.84-0.92, *P* = 2.98E-7), 8% decreased risk of MDD (OR = 0.92, 95% CI = 0.90-0.95, *P* = 4.96E-12), and 11% decreased risk of BIP (OR = 0.89, 95% CI = 0.87-0.91, *P* = 2.88E-12). Additionally, two IgG N-glycosylation traits IGP34 and IGP57 were causally associated with an increased risk of SCZ, each SD increase in genetically determined IGP34 and IGP57 was associated with a 13% (OR = 1.13, 95% CI = 1.09-1.17, *P* = 7.37E-13) and 10% (OR = 1.1, 95% CI = 1.07-1.12, *P* = 1.55E-12) increased risk of SCZ. In contrast, IGP34 and IGP57 were not associated with MDD or BIP (**Supplementary Table S4**).

We observed a significant causal association between genetically predicted IgG N-glycosylation trait IGP21 and the risk of PTSD (**Figure 2**). Each SD increase in genetically determined IGP21 was associated with a 9% increased risk of PTSD (OR = 1.09, 95% CI = 1.04-1.14, *P* = 8.67E-5). For ADHD, IGP22 was associated with a reduced risk (OR = 0.95, 95% CI = 0.93-0.96, *P* = 1.43E-10), with each SD increase in genetically determined IGP22 associated with a 5% decrease in the risk of ADHD. Additionally, no significant causal relationship between IgG N-glycosylation traits and ASD was observed (**Supplementary Table S4**).

### 3.3 The causal effect of psychiatric disorders on IgG N-glycosylation traits

To assess the potential causal influence of psychiatric disorders on IgG N-glycosylation traits, we employed the IVW method as the primary MR approach. Because there were not enough genome-wide significant SNPs for PTSD, and ASD, we only analyzed the causal effects of SCZ, MDD, BIP, and ADHD on IgG N-glycosylation traits. We did not detect significant causal effects of four psychiatric disorders on the IgG N-glycosylation traits (**Supplementary Table S5**).

### 3.4 Sensitivity analyses

We performed sensitivity analyses to validate our putative causalities obtained with bidirectional MR. First, the results of Cochran’s Q test showed no obvious heterogeneity for all significant associations (**Supplementary Table S6, 7**). Second, leave-one-out analyses showed the causal effect was not driven by a single instrumental variable (**Supplementary Table S8, 9**). Third, MR-Egger intercepts for all associations were in proximity to zero, indicating the absence of significant pleiotropy detection (**Supplementary Table S10, 11**). Fourth, the global MR pleiotropy residual sum and outlier (MR-PRESSO) test did not detect any evidence of horizontal pleiotropy (**Supplementary Table S12, 13**). Overall, the sensitivity analyses confirmed the reliability of our putative causal effects in both the forward and reverse MR results.

## 4 Discussion

Elucidating the causal links between IgG N-glycosylation traits and psychiatric disorders could greatly enhance our understanding of their pathogenesis. We conducted a bidirectional MR analysis to assess the causal associations between IgG N-glycosylation traits and the risk of psychiatric disorders including SCZ, MDD, BIP, PTSD, ADHD, and ASD in European populations. Our robust evidence strongly indicates causal relationships between IgG N-glycosylation traits and SCZ, MDD, BIP, PTSD, and ADHD, whereas no significant link with ASD is observed.

A compelling example of the pathogenic influence of the immune system on brain function is the change in mood, social behavior and cognitive abilities — known as sickness behavior — upon infection and systemic inflammation. The release of pro-inflammatory cytokines, affects the brain via neural (mainly vagal) pathways, interaction with cytokine receptors on cerebral endothelial cells and/or microglial activation (48). Various psychiatric disorders have been verified to be associated with inflammation. IgG is an antibody integral to the adaptive immune system, which has evolved to safeguard the human species against pathogens and has a key role in various immune processes. IgG glycans serve as a critical determinant, acting as a switch between the pro- and anti-inflammatory states of the IgG molecule (14). They possess the capability to guide the immune response elicited by this antibody (49). Although some MR studies have reported causal relationships between systemic inflammatory regulators and circulating proteins with psychiatric disorders, they do not investigate the role of IgG N-glycosylation (50, 51). This study establishes causal connections between distinct IgG N-glycosylation patterns and various psychiatric disorders, highlighting their potential biomarkers.

Our findings suggest that an elevated level of the IgG N-glycosylation trait IGP22 represents a decreased risk of ADHD. IGP22 serves as a direct metric for IgG N-glycans, primarily modified by the three sugar molecules: fucoses, galactoses, and sialic acids. In a recent study, there was no significant difference in IGP22 levels between patients with ADHD and control group, likely due to a small sample size. However, a statistically significant decrease was observed in sialylated IgG levels within the ADHD sample, as well as in chronic inflammatory and autoimmune diseases (17, 52), suggesting the pivotal role of IgG sialyation in patients. Notably, IGP22 differs from IGP17 by the addition of a sialic acid molecule, and IGP17 exhibited a statistically significant decline (23). Moreover, published data show that IgG sialyation exhibits an anti-inflammatory activity (53). Numerous studies on its mechanism have shown that its anti-inflammatory activity relies on the inhibitory receptor FcγRIIB (54). Patients with chronic neuroinflammation undergoing intravenous immunoglobulin therapy with α-2,6 sialylated IgG infusion exhibit an enhanced surface expression of FcγRIIB, leading to a reduction in inflammatory response (55–57). Based on the observations, we hypothesize that the sialylation of IgG plays a pivotal role in exerting anti-inflammatory effects by enhancing the surface expression of FcγRIIB, thereby underlying the pathogenesis of ADHD. However, further works are necessary to validate the assertion and elucidate the precise mechanisms involved.

Genetically predicted elevation in IGP7, an IgG N-glycosylation trait directly measured and modulated by fucose and galactose, appears protective against MDD, BIP, and SCZ. These findings suggest potential shared pathophysiological mechanisms among these psychiatric conditions, corroborating studies that have shown overlapping etiologies in these three psychiatric disorders (58). A study has shown that the IgG4- galactose trait is correlated with the severity of MDD patients (20), which is inconsistent with our study. On the other hand, another study has demonstrated a significant decrease in the level of GP13 (which differs from GP7 by a galactose molecule) in the high abundance serum proteins fraction of serum from female patients with SCZ (19). These contradictory findings suggest that the galactose molecule plays a crucial role in both disorders. The presence or absence of additional Gal residues at the glycan arms has been controversially linked to altered affinities to diverse IgG effector structures. Depending on the circumstances, galactosylation can either strengthen or weaken the affinity of IgG for FcR, thereby modulating its functional properties (14). Nevertheless, one consistent observation is that the presence of additional galactose molecules at the glycan arms of IgG seems to exert a crucial influence on its functional characteristics and is associated with promoting anti-inflammatory processes. Notably, patients with autoimmune diseases like rheumatoid arthritis (RA) and lupus erythematosus (LE) exhibit elevated levels of agalactosylated total IgG, suggesting the importance of galactose in regulating IgG function in these conditions (59, 60). Future research on IgG N-glycosylation traits in relation to MDD, and SCZ, should particularly focus on the galactosylation of IgG.

However, existing research indicates that MDD, ASD, SCZ, and BIP share genetic risk factors (61), while no significant causal relationship between ASD and IgG N-glycosylation traits. There may be some reasons as following. First, the statistical power of the available ASD GWAS data may be lower compared to other disorders, which could limit the ability to detect significant associations. Second, ASD is highly heterogeneous, both genetically and phenotypically, which may dilute potential causal signals. Additionally, the effects of IgG N-glycosylation on ASD risk may be more specific to certain tissues or developmental stages that were not fully captured in our analysis. The biological mechanisms underlying ASD, particularly in terms of immune or neurodevelopmental pathways, may differ from those of other psychiatric disorders such as MDD, SCZ, and BIP.

Conversely, the elevated levels of IGP34 and IGP57 are associated with an increased risk of SCZ and are not observed in the MDD and BIP. Current observation has not directly examined the relationship between these glycosylation traits and SCZ, and the lack of direct evidence prompts us to explore potential underlying mechanisms by analyzing the similarities and differences between IGP34 and IGP57. IGP34 is characterized by the ratio of fucosylated (without bisecting GlcNAc) monosialylated and disialylated structures in total IgG glycans, while IGP57 is characterized by the percentage of digalactosylated structures in total neutral IgG glycans. The fact that IGP34 and IGP57 possess completely different structures suggests that specific IgG N-glycosylation structures can exert similar functional roles. Multiple studies have shown that eliminating the core fucose residue enhances the binding affinity of IgG to the human FcγRIIIA receptor, subsequently potentiating antibody-dependent cell-mediated cytotoxicity (ADCC) activity (62, 63). A study on COVID-19 patients revealed an association between increased total IgG fucosylation and with severity of COVID-19 (64). Based on what we discussed above of galactosylation and sialylation, these indicate that various glycosylation molecules ultimately exert pro-inflammatory effects and suggest a pro-inflammatory role of IgG fucosylation in the development of schizophrenia.

A directly measured IgG N-glycan, IGP21, is found to be associated with an elevated risk of developing PTSD. In one study on PTSD, IgG N-glycosylation traits do not exhibit notable variations (22). In this study, the level of IGP21 in PTSD patients was higher than the control, but had no statistical meaning, which may be due to small samples or influenced by other confounding factors. However, the IGP22 level was significantly lower in PTSD patients (22). IGP21 is characterized by bisecting GlcNAc, galactose, and sialic acid. The IGP22 differs from IGP21 by adding fucose. These indicate the fucosylation of IgG may play an important role in the development of PTSD. Core fucosylated N-glycans on IgG are generally considered pro-inflammatory as core fucosylation of IgG can drastically decrease ADCC (65). Theoretically, IGP22 should have an increased risk of PTSD according to our results, but the results of Lucija Tudor’s study is the opposite (22). As we discussed above, we have focused on the role of bisecting GlcNAc. In some chronic inflammatory diseases, such as systemic LE, patients exhibit upregulated levels of bisecting GlcNAc (66). Whereas in RA, it seems to have minimal or no effect (67). These variations highlight the intricate and diverse roles of bisecting GlcNAc across different inflammatory conditions. Additionally, elevated levels of bisecting GlcNAc glycans have been documented in immune diseases, including Alzheimer’s (AD) (68), ischemic stroke (69), dementia (70). Significantly, There were some studies indicated IgG exerts an anti-inflammatory effect when the bound *N*-glycan contains galactose, sialic acid or fucose, while the addition of bisecting GlcNAc has a proinflammatory effect (71, 72). Both underscore the vital role of bisecting GlcNAc of promoting inflammation when different sugar molecules exist in IgG. Future research on IgG N-glycosylation traits in relation to PTSD should particularly focus on the bisecting GlcNAc of IgG.

In this study, we offer new insights and directions for psychiatrists dedicated to research on inflammation and psychiatric disorders. Our study emphasizes the potential impact of different IgG glycosylation patterns on psychiatric disorders and the resultant pro-inflammatory or anti-inflammatory effects when multiple glycan molecules are combined. Therefore, it is particularly important to regulate glycan molecules that play a crucial role in the pathogenesis of psychiatric disorders. Glycoengineering, which involves changing the glycosylation patterns of proteins, is therefore expected to be an effective means of overcoming the problems of therapeutic proteins (73). Modifying the glycan composition to achieve anti-inflammatory effects or to reduce the pro-inflammatory actions of these molecules may be a promising approach for the treatment of psychiatric disorders.

This study’s key strengths are primarily derived from the utilization of the MR design, which effectively mitigates several inherent limitations associated with observational studies. Additionally, we systematically investigated the connections between IgG N-glycosylation traits and psychiatric disorders using summary-level data from extensive genetic consortia and cohorts. Another noteworthy strength is the consistency observed across various MR approaches, underscoring the robustness of our findings.

It is crucial to acknowledge several limitations in our study. Firstly, our works are confined to individuals of European ancestry, potentially limiting the applicability of our findings to other populations, including Asian or African ancestry. Secondly, the data itself may have inherent limitations, meaning that conclusions drawn from it can only be considered reliable after experimental validation. Our analysis aims to provide insights and direction for future research. Thirdly, the discussion on the positive impacts of IgG N-glycosylation traits on BIP is limited due to the lack of relevant research. Forth, although we have used very strict criteria to eliminate confounding factors, there are still unknown confounding factors that affect our results.

## 5 Conclusions

This study revealed the causal relationship between distinct IgG N-glycosylation traits and multiple psychiatric disorders. Notably, IGP7 may play a protective role in the development of SCZ, MDD, BIP. Certain disorders share a causal link with identical glycosylation traits, indicating a common underlying etiology among these conditions. These findings highlight the potential of IgG N-glycosylation patterns as predictive biomarkers for psychiatric disorders. These discoveries call for further investigation to visualize the underlying mechanisms, offering the potential to guide more personalized diagnostic and therapeutic strategies in the management of psychiatric disorders.

## Data Availability

The datasets presented in this study can be found in online repositories. Data on the IgG N-glycosylation traits were available at https://doi.org/10.7488/ds/2481. Data on psychiatric disorders were downloaded from the PGC (https://www.med.unc.edu/pgc/download-results/). Our analysis code has been uploaded into the github (https://github.com/chun-123/IgG-N-glycosylation-psychiatric-disorders). Further inquiries can be directed to the corresponding author/s.
